# Defining the methanogenic SECIS element *in vivo* by targeted mutagenesis

**DOI:** 10.1080/15476286.2025.2472448

**Published:** 2025-02-25

**Authors:** Nils Peiter, Anna Einert, Pauline Just, Frida Jannasch, Marija Najdovska, Michael Rother

**Affiliations:** Fakultät Biologie, Technische Universität Dresden, Dresden, Germany

**Keywords:** Archaea, *Methanococcus*, reporter, SECIS element, selenocysteine

## Abstract

In all domains of life, Archaea, Eukarya and Bacteria, the unusual amino acid selenocysteine (Sec) is co-translationally incorporated into proteins by recoding a UGA stop codon to a sense codon. A secondary structure on the mRNA, the selenocysteine insertion sequence (SECIS), is required, but its position, secondary structure and binding partner(s) are not conserved across the tree of life. Thus far, the nature of archaeal SECIS elements has been derived mainly from sequence analyses. A recently developed *in vivo* reporter system was used to study the structure–function relationships of SECIS elements in *Methanococcus maripaludis*. Through targeted mutagenesis, we defined the minimal functional SECIS element, the parts of the SECIS where structure and not the identity of the bases are relevant for function, and identified two conserved -and invariant- adenines that are most likely to interact with the other factor(s) of the Sec recoding machinery. Finally, we demonstrated the functionality of SECIS elements in the 5`-untranslated region of the mRNA and identified a potential mechanism of SECIS repositioning in the vicinity of the UGA for efficient selenocysteine insertion.

## Introduction

Members from all domains of life incorporate selenium into proteins, such as selenocysteine (Sec), the 21^st^ amino acid [1,2]. Most known Sec-containing proteins (selenoproteins) are oxidoreductases. In mammals, selenoproteins are mostly involved in redox homoeostasis and antioxidant activity. In methanogenic Archaea, they are mainly involved in the primary energy metabolism, methanogenesis [3]. One potential explanation for the presence of Sec in the catalytic site centre is the lower reactivity of cysteine (Cys) exhibited by the higher pK_a_ of the protonated thiol group than that of the deprotonated selenol group of selenocysteine under physiological conditions [4]. Another possible reason could be the broader range of substrates and microenvironmental conditions for optimal enzyme activity [5]. Sec synthesis is conserved in all three domains and occurs in a tRNA-bound manner. However, specific aspects of the respective mechanisms differ. A common feature is the ‘mischarging’ of the Sec-specific tRNA tRNA^Sec^ with serine. Subsequently, eukaryotes and archaea spend an additional ATP for the formation of a phosphorylated seryl-tRNA^Sec^ intermediate [6,7].

**Figure 1.**
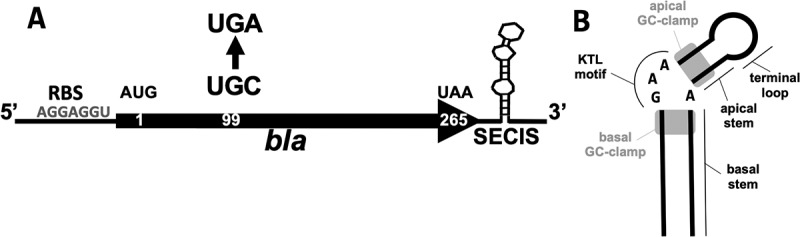
General scheme of the beta-lactamase (Bla) reporter construct.

The stop codon UGA on the mRNA encoding selenoproteins designates co-translational Sec incorporation (instead of translation termination) when a specific secondary structure on the mRNA, the Sec insertion sequence (SECIS) element, is also present on the mRNA [8–10]. However, their location, sequence and structure differ across the domains of life. In eukaryotes and archaea, SECIS elements are found (with one exception) in the 3' untranslated region (UTR) of the selenoprotein mRNA [8,11]. In bacteria, they are directly located in 3 of the UGA in the open reading frame [12]. Sec incorporation involves a specialized translation elongation factor, SelB (eEFsec in eukaryotes) [13–15]. In bacteria, SelB binds to both Sec-tRNA^Sec^ and SECIS, thereby ‘directly’ mediating communication between the recoding site (UGA at the A site of the ribosome) and the recoding signal (SECIS) [16]. In eukaryotes, an additional protein, SECIS binding protein 2 (SBP2), is involved, and the structure of the ‘selenosome’ complex, comprising eEFsec-bound Sec-tRNA^Sec^, SBP2 and SECIS bound to the ribosome, has recently been elucidated [17]. Eukaryotic SECIS elements contain two conserved structural features. One of these is bound by eEFsec, whereas the other is bound by SBP2 [17]. Notably, no homologue of SBP2 was identified in archaea [18], and archaeal SelB could not bind to a cognate SECIS [15], leaving the details of the mechanism for Sec insertion unknown. In Lokiarchaea, SECIS elements (identified bioinformatically) exhibit conserved features that resemble those of eukaryotes [18]. The study concluded that Lokiarchaea could represent a transitional state for the UGA recoding mechanisms between Archaea and Eukarya. Recently, it was argued that within the Archaea, methanogenic SECIS elements might have originated from those of Asgard Archaea [19]. Canonical archaeal SECIS elements were initially identified almost exclusively in the 3'-UTR of selenoprotein mRNAs in methanogenic members of this domain, through the presence of a single conserved motif, an internal asymmetric loop comprising GAA and an opposite A [11] (Fig. 1). As it is reminiscent of the conserved kink-turn motif found in eukaryotic SECIS elements, it was designated as a kink-turn-like (KTL) motif [20]. This motif is usually flanked by two G-C Watson and Crick base-pairing clamps. The bases of the terminal loop and the basal stem appear not to be conserved. Notable exceptions include the SECIS element of the *fwuB* gene in Methanococci containing an AAA and opposite A and that of the *fdhA1* gene, which appears to be located in the 5'-UTR.

(A) RBS, ribosome binding site in the 5'-UTR of the *sla* promoter; AUG, start codon; UAA, stop codon; arrow, codon exchange of UGC to UGA. The numbers within the coding region of *bla* indicate the amino acid position of the translated protein; the SECIS element variants are placed 3' of the coding sequence of *bla*. This figure was adopted from Ref [20]. (B) Schematic of SECIS element in methanogenic Archaea. The presentation of the schematic is based on data from Ref [18]. For an explanation of the structural motifs, see text.

The *in vivo* function of archaeal SECIS elements could be analysed by following insertion of radiolabelled selenium into the respective selenoprotein, a rather expensive and tedious procedure [10], until a convenient genetic reporter system was developed for *Methanococcus maripaludis* JJ [20] (Figure 1A), an archaeal model organism for the study of selenium biology [21–23]. The *Escherichia coli* gene for beta-lactamase (*bla*) was engineered into a selenoprotein gene under the control of a constitutive promoter by exchanging a non-catalytic Cys codon to a Sec codon and adding the SECIS element of the *fruA* gene (encoding the large, Sec-containing, subunit of F_420_-reducing hydrogenase) of *M. maripaludis* JJ downstream of the coding region (Figure 1A). Assessing Bla activity using the chromogenic substrate nitrocefin in lysates of *M. maripaludis* strains carrying this translational reporter on a self-replicating plasmid established direct selenium and SECIS dependence of Sec-containing Bla activity, quantified in lysates [20] (Figure 1A). Using this reporter system, we showed that disrupting the structural integrity of the KTL motif (Figure 1B) through the exchange of a single base in the *fruA* SECIS element completely abolished its function [20].

In this study, we extended the structure–function analysis of *M. maripaludis* SECIS elements by assessing the relevance of individual structural elements of *fruA* and by characterizing the SECIS elements of other selenoprotein genes. Critically, the functionality of a SECIS element uniquely located in the 5-untranslated region of the mRNA was also revealed.

## Results

### Sec insertion into Bla is not affected by the synthesis of other selenoproteins

**Figure 2.**
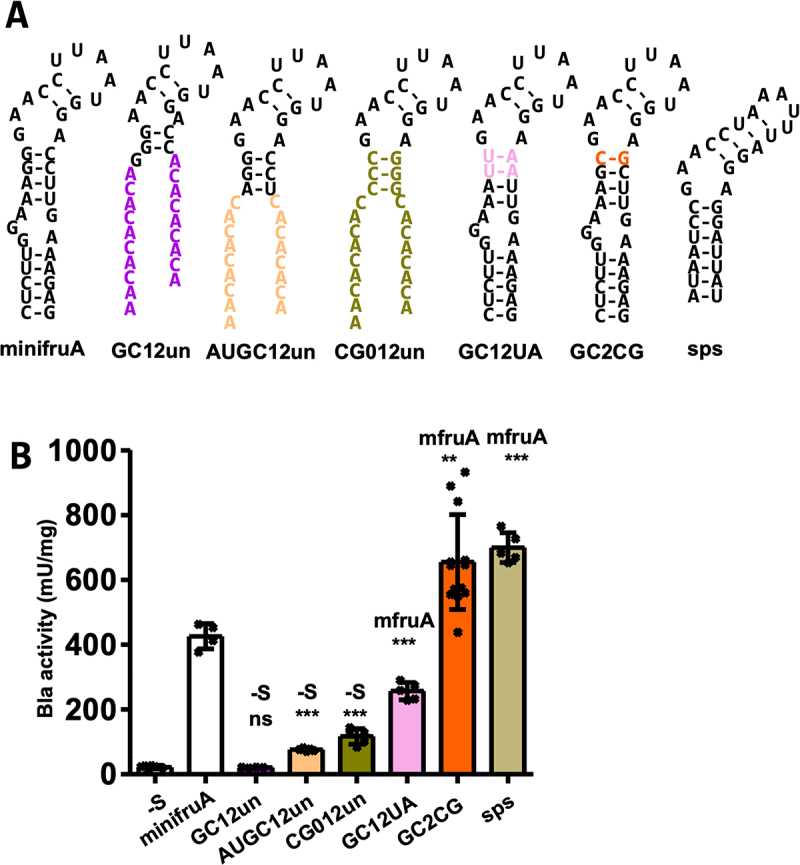
Impact of base exchanges in the basal stem of the methanogenic SECIS element.

*M. maripaludis* JJ contains nine selenoproteins, at least five of which are involved in energy metabolism [24]. For the quantitative assessment of selenoprotein synthesis, a previously engineered Bla reporter [20] (Figure 1A) was employed. The starting (and reference) point for our structure–function probing of methanogenic SECIS elements in *M. maripaludis* was the construct minifruA, which contained the minimized 3'-UTR of the *fruA* SECIS element (Figure 2A). The first question we addressed was whether the capacity of the Sec synthesis and insertion machinery is a limiting factor for the synthesis of Sec-containing Bla. To this end, the synthesis of Sec-containing Bla in the wild type (strain JJ) was compared to that in a strain that can only synthesize one endogenous selenoprotein, FdhA (*hrsM* disruption strain JH7 [23]). Strain JH7 carrying the minifruA reporter displayed Bla activity of 368 ± 39 mU/mg, which was not higher than that observed in the wild type (426 ± 47 mU mg^1^ [20]), strongly suggesting that the capacity of the Sec synthesis and insertion machinery is not a limiting factor for Bla activity.

(A) Predicted secondary structures of SECIS variants assessed in (B). Minimum free energy RNA structures were predicted using RNAfold [25]; hyphens indicate Watson–Crick base pairing, and coloured letters indicate nucleotide exchange compared to minifruA SECIS (Figure 2A). (B) Specific activity of Bla in cleared cell lysates of *M. maripaludis* carrying different variants of minifruA SECIS element or putative SECIS element of *M. maripaludis* JJ selenophosphate synthetase gene (*sps*), specific activities for the minifruA SECIS and the no-SECIS control (-S) are from [20]. Error bars show the standard deviation of at least four biological replicates; crosses indicate individual data points; the experiment was reproduced at least once; comparison was performed either with minifruA (mfruA) or -S using an unpaired two-tailed t-test. *, *p* < 0.05; **, *p* < 0.01; ***, *p* < 0.001.

### Probing the basal stem of the methanogenic SECIS element

For structure–function probing, the reference SECIS (minifruA) was replaced with its variants or SECIS elements from other selenoprotein genes. Their relative functionality was assessed by quantifying Bla activity in cleared lysates of *M. maripaludis* strains carrying the respective construct on a self-replicating vector. Preceding (from the stem-loop base, Figure 1B), the conserved KTL motif is a stem of variable length and composition [3]. First, a SECIS element was tested, in which all bases distal to the basal GC clamp were altered to prevent the formation of a duplex, thereby eliminating most of the stem. (GC12un, Figure 2A). The corresponding Bla activity (21 ± 2 mU mg^−1^) was not significantly different from that of the construct lacking a SECIS element (-S, 22 ± 5 mU mg^−1^, Figure 2B), suggesting that the remaining SECIS in GC12un was not functional *in vivo*. Prediction of the RNA structure suggested that the basal GC clamp alone is insufficient for stable preservation of the KTL motif, which is the most highly conserved and previously demonstrated to be essential for SECIS function [20]. Consistent with this interpretation, the addition of a single Watson and Crick base pair (A-U) basal to the GC clamp increased the Bla activity by approximately 3-fold (AUGC12un, 76 ± 4 mU mg^−1^, Figure 2B). To further increase the stability (Table S1), the basal GC clamp was further extended by one base pair and inverted (to C-G base pairs). The resulting Bla activity was also increased compared to AUGC12un, if only moderately (GC012un, 117 ± 24 mU mg^1^, Figure 2B). Therefore, a three base pair basal stem (AUGC12un and CG012unpair variants, Figure 2A) appears to represent the ‘shortest’ required structure for a functional methanogenic archaeal SECIS element. Replacing the basal GC clamp with weaker U-A base pairs (GC12UA variant, Figure 2A) reduced Bla activity by approximately 40% (257 ± 27 mU mg^−1^, Figure 2B) compared to that of minifruA (Figure 2B). It appears that the base pair identity in this region of the SECIS is not the relevant determinant for its function, but the extent to which it stabilizes the KTL motif. Consequently, when G**·**A (non-Watson–Crick) base pairing between the bulged A of the KTL motif and the opposite G of the base pair of the GC clamp was restricted, achieved by simply switching it to a C-G base pair (GC2CG variant, Figure 2A), Bla activity increased by as much as 50% (655 ± 147 mU mg^−1^, Figure 2B) compared to that of minifruA. Similar Bla activity was observed when the SECIS element of the selenophosphate synthetase gene (*sps*) was placed in the 3'-UTR of the reporter (sps, 699 ± 46 mU mg^−1^, Figure 2B), which naturally contains a basal GC clamp of two C-G base pairs (Figure 2A). This constellation may confer a beneficial effect on the functionality of the SECIS element by conserving the kink-turn motif.

### Probing the apical stem and the terminal loop of the methanogenic SECIS element

**Figure 3.**
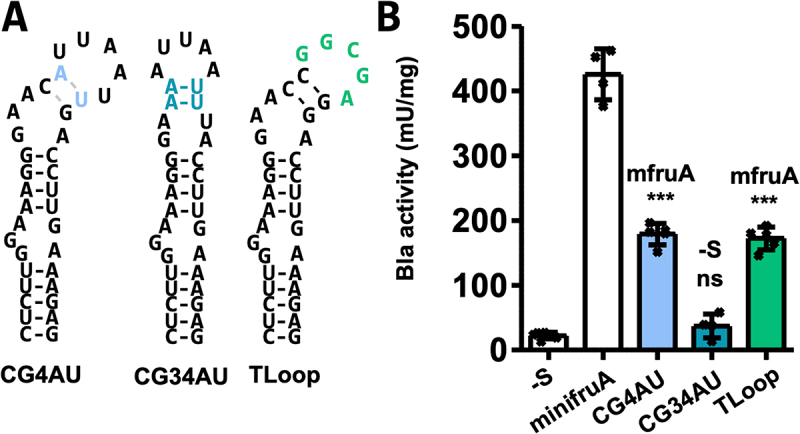
Impact of base exchanges in the apical stem and terminal loop of the methanogenic SECIS element.

Unlike in other (putative) archaeal SECIS elements (e.g. sps, Figure 2A), the apical (relative to the KTL motif) stem of the *fruA* SECIS element of *M. maripaludis* consists only of two C-G base pairs, referred to here as the ‘apical GC clamp’ (Figure 1B). Exchanging the distal (relative to the KTL motif) C-G base pair of the apical GC clamp to an A-U base pair (CG4AU, Figure 3A) resulted in a reduction in Bla activity by approximately 60% (179 ± 16 mU mg^−1^, Figure 3B) compared to that of minifruA (Table S2). RNA structure prediction suggested that the apical GC clamp with the distal C-G changed to A-U is likely unpaired (Figure 3A), which may be true for approximately half of the population of SECIS elements *in vivo* (Table S1). Changing both C-G base pairs of the apical GC clamp to A-U base pairs (CG34AU, Figure 3A) resulted in the complete loss of function of the SECIS element (Figure 3B, Table S2). This finding was again consistent with the RNA structure prediction, which indicated that the structure of the KTL motif was similarly disrupted as in construct GC12un (Figure 2A). Structural predictions for the exchange of the proximal C-G base pair to A-U claim the same weakening of the apical GC clamp and thus lack of complete conservation of the KTL motif as in CG4AU and were therefore not further pursued. Together, these results indicate that the apical GC clamp of the *fruA* SECIS already attains a ‘minimal’ configuration, leading to substantial impairment of its functionality if altered. The SECIS from *sps* had a longer apical stem (Figure 2A). Given that the GC2CG SECIS and the natural SECIS from *sps* have apical stems of different lengths but exhibit similar recoding efficiencies, it may be argued that the extension of the apical stem exerts only a minor, if any, impact on the functionality of the SECIS element.

(A) Predicted secondary structures of SECIS variants assessed in (B). Minimum free energy RNA structures were predicted using RNAfold [25]; hyphens indicate Watson–Crick base pairing, and coloured letters indicate nucleotide exchange compared to minifruA SECIS (Figure 2A). (B) Specific activity of Bla in cleared cell lysates of *M. maripaludis* carrying different variants of minifruA SECIS element, specific activities for minifruA SECIS and the no SECIS control (-S) are from [20]. Error bars show the standard deviation of at least four biological replicates; crosses indicate individual data points; the experiment was reproduced at least once; comparison was performed either with minifruA (mfruA) or -S using an unpaired two-tailed t-test. ns, not significant; ***p < 0.001.

The terminal loop in methanoarchaeal SECIS elements comprises four to six bases, none of which is conserved [3,26]. The five terminal loop bases of *fruA* were subjected to a change in their GC content, and the introduction of new base pairing was avoided. (TLoop, Figure 3A). Compared to minifruA (Table S2), an approximately two-fold reduction in Bla activity was observed (172 ± 18 mU mg ^1^, Figure 3B). Although not conserved, the bases comprising the loop structure in methanogenic SECIS elements are predominantly A and U [18]. The observation that the TLoop variant is comprised mainly of G and C but still reasonably active as SECIS element suggests that the loop bases are not very relevant for SECIS functionality.

### Probing the conserved KTL motif

**Figure 4.**
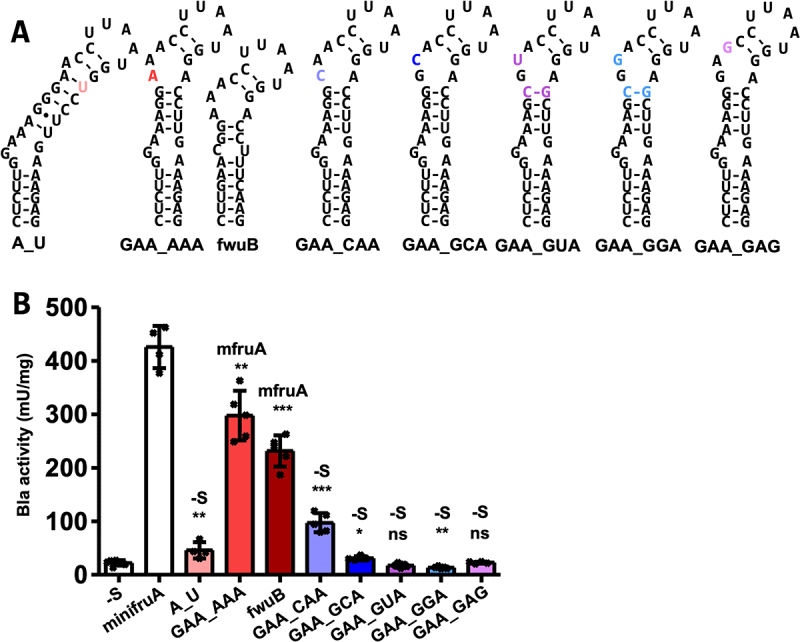
Impact of base exchanges in the KTL motif of the methanogenic SECIS element.

We previously showed that changing the opposing A in the KTL motif (Figure 1B) to C resulted in a complete loss of functionality as a SECIS [20]. The KTL motif of this variant was predicted to be disrupted. Disruption of the KTL motif was also predicted for a corresponding A → U variant (A_U, Figure 4A), which also almost completely lacked SECIS functionality (46 ± 15 mU mg^−1^, Figure 4B). An A → G variant (of *M. jannaschii fruA*), predicted to contain an intact KTL motif, retained some functionality as SECIS [10], again arguing that structure, rather than base identity, is relevant for function. Therefore, we aimed to ascertain the consequences of base changes within the KTL motif without disrupting its (predicted) structure. Substituting G for A in the KTL motif (GAA_AAA, Figure 4A) resulted in only a moderate reduction in Bla activity (298 ± 42 mU mg^−1^, Figure 4B) compared to that of minifruA (Table S2). Notably, such a KTL motif variant occurs naturally in the predicted SECIS element for *fwuB*, which encodes the Sec-containing subunit of formylmethanofuran dehydrogenase [3]. Using this SECIS element to direct Sec insertion into Bla (fwuB, Figure 4A) resulted in approximately half of the activity (232 ± 29 mU mg^−1^, Figure 4B) compared to that of minifruA (Table S2). The AAA/A KTL containing SECIS is therefore active but has a lower recoding capacity. The exchange of G in the KTL motif to C, predicted to retain its structure (GAA_CAA, Figure 4A), was also still active as SECIS but fourfold reduced (97 ± 18 mU mg^−1^, Figure 4B) compared to that of minifruA (Table S2). These results suggest that this position exhibits some arbitrariness in terms of the identity of the base, that is, it is not essential for functionality.

(A) Predicted secondary structures of SECIS variants assessed in (B). Minimum free energy RNA structures were predicted using RNAfold [25]; hyphens indicate Watson–Crick base pairing, and coloured letters indicate nucleotide exchange compared to minifruA SECIS (Figure 2A). (B) Specific activity of Bla in cleared cell lysates of *M. maripaludis* carrying different variants of minifruA SECIS element, specific activities for minifruA SECIS and the no SECIS control (-S) are from [20]. Error bars show the standard deviation of at least four biological replicates; crosses indicate individual data points; the experiment was reproduced at least once; comparison was performed either with minifruA (mfruA) or -S using an unpaired two-tailed t-test. ns, not significant; *, *p* < 0.05; **, *p* < 0.01 ***; *p* < 0.001.

This ambiguity was not observed for the two A residues in the GAA sequence of the KTL motif. When the first A was changed to C (GAA_GCA), U (GAA_GUA) and G (GAA_GGA) (Figure 4A), the corresponding Bla activities were similar to those in the no-SECIS background (31 ± 4 mU mg^−1^, 18 ± 4 mU mg^−1^, and 14 ± 2 mU mg^−1^, respectively, vs 22 ± 5 mU mg^−1^, Table S2). A similar result was obtained when the second A of the GAA in the KTL motif was changed to G (GAA_GAG, 23 ± 2 mU mg^−1^). None of the four GAA variants exhibited discernible structural alterations in Minimum Free Energy (MFE) structural prediction (Figure 4A). However, these single-base exchanges resulted in a complete loss of function as SECIS (Figure 4B). Unlike the other positions probed, these two residues have to be A to yield a functional SECIS, which is indicative of (a) specific molecular interaction(s) essential for the recoding mechanism.

### The methanogenic SECIS element in the 5-UTR

**Figure 5.**
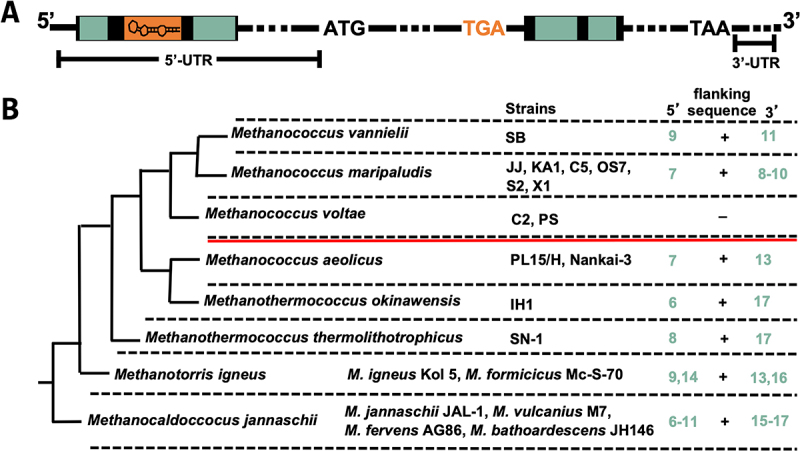
Abundance of archaeal SECIS elements in the 5-UTR of *fdh* flanked by coding sequence.

The only SECIS element of methanogenic archaea predicted to be located in the 5'-UTR of the mRNA is that of *fdhA1*, which encodes a Sec-containing subunit of formate dehydrogenase [3,11]. We noticed that the SECIS element of *fdhA1* was naturally flanked by two sequences, seven (5') and ten (3’) bases, which are complementary to a sequence within the downstream open reading frame of *fdhA1* (Figure 5A). Assuming duplex formation of the complementary sequences, the SECIS element would be localized at 43 nt 3' of the UGA Sec codon. This phenomenon is not restricted to *M. maripaludis* JJ but instead appears to be conserved in Methanococcales (Figure 5B). Furthermore, the higher the growth temperature, the longer the flanking sequences appear to be, as observed for the thermophilic clades *Methanothermococcus, Methanotorris* and *Methanocaldococcus* (Figure 5B). Notably, *Methanococcus voltae* appears to lack a SECIS element in the 5'-UTR of *fdhA*, and with it, the flanking complementary sequences.

(A) General scheme of the gene locus encoding formate dehydrogenase subunit A (*fdhA1*) in methanogenic archaea. ATG, translational start codon of *fdhA1*; TGA, Sec codon; TAA, stop codon; orange box, sequence of the SECIS element; green box, complementary sequences; dotted line, intermittent sequence. (B) Maximum-likelihood phylogenetic tree of Methanococcales species adapted from [27], based on 16S rRNA gene sequences. The presence (+) or absence (–) of sequences complementary to the coding region flanking the SECIS element in different organisms and strains of Methanococcales and numbers in green indicate the maximum and minimum number of complementary nucleotides to the flanking sequence of the SECIS element for each species 5' or 3' of the SECIS. The red line indicates the evolutionary transition from thermophilic to mesophilic growth.

**Figure 6.**
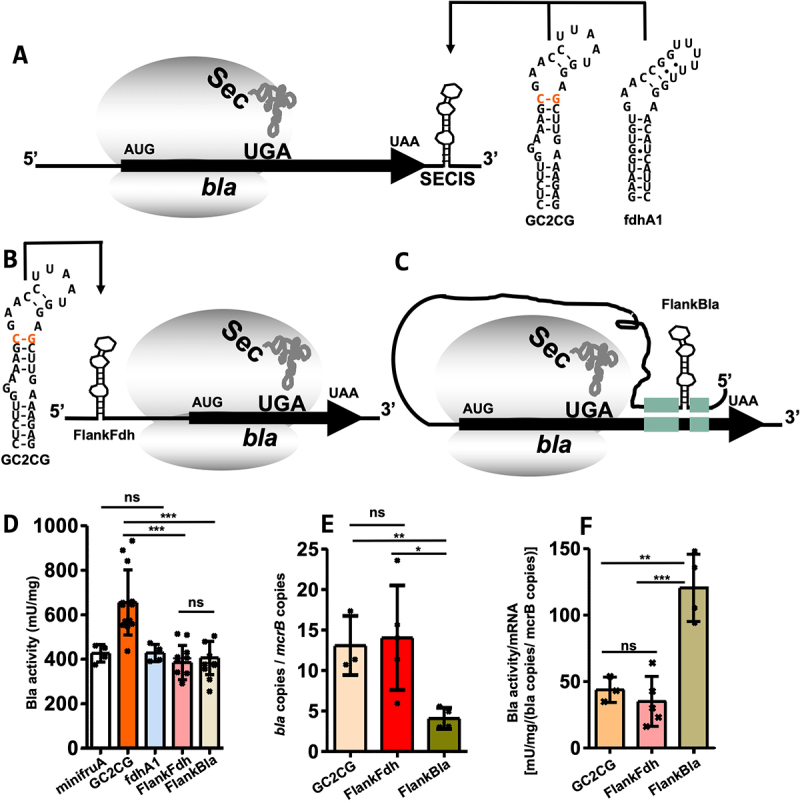
SECIS elements in the 5'-UTR.

Whether the predicted structure in the 5'-UTR of *fdhA1* functions as SECIS was assessed by placing it in the deduced 3'-UTR of the Bla reporter (fdhA1, Figure 6A). Bla activity (428 ± 39 mU mg^−1^, Figure 6D) in the strain carrying the corresponding construct demonstrated that the element is indeed a functional SECIS element. To experimentally approach both the issue of SECIS element location in the 5'-UTR and of flanking sequences complementary to the coding region, the GC2CG variant (the ‘strongest’ SECIS available to us) was flanked with the sequences flanking *fdhA1* SECIS (i.e. not complementary to sequences of *bla*) and the deduced 5'-UTR of the *bla* reporter gene (FlankFdh, Figure 6B). The resulting Bla activity (385 ± 77 mU mg^−1^, Figure 6D) was only slightly lower than that of the 3'-UTR GC2CG SECIS variant (Figures 2B and 6A, Table S2). To the best of our knowledge, this is the first experimental demonstration of Sec insertion guided by a SECIS element in the 5'-UTR of any selenoprotein mRNA. The results also show that, at least in our reporter system, positioning the 5'-SECIS in the vicinity of the UGA is not required. Whether such potential positioning has an effect was tested by flanking the GC2CG SECIS with sequences complementary to *bla*, potentially positioning it at 43 nt 3' of the UGA and placing the construct in the 5'-UTR of the Bla reporter (FlankBla, Figure 6C). The resulting Bla activity was not significantly different from that of the construct without sequences complementary to *bla* (405 ± 75 mU mg^−1^, Figure 6D).

Scheme of SECIS location on the mRNA of the reporter construct (A–C). UGA indicates Sec codon; Sec, selenocysteine bound to tRNA^Sec^; black arrow, ORF *bla*; circles, small and large subunits of the ribosome; SECIS element variant (schematic secondary structure) is placed in the 3'-UTR or 5'-UTR of the coding sequence within the *sla* promoter region. (A) Predicted secondary structure of *fdhA1* SECIS element and GC2CG (see Figure 2A); thin black arrow indicates either one of the constructs is tested in the 3'-UTR (or 5'-UTR, FlankFdh, B). Minimum free energy RNA structures were predicted using RNAfold [25]; hyphens indicate Watson–Crick base pairing; dots indicate non-Watson–Crick base pairing. (C) Possible SECIS relocation for SECIS elements in the 5'-UTR (C, FlankBla); green bars indicate relocation with complementary sequences. (D) Specific activity of Bla in cleared cell lysates of *M. maripaludis* carrying reporter constructs either *fdhA1* SECIS in the 3'-UTR of the reporter or constructs GC2CG in the 5'-UTR with (FlankBla) or without (FlankFdh) complementary sequences of *bla* ORF; data for GC2CG and minifruA are shown also in Figure 2. (E) Abundance of *bla* mRNA. Samples correspond to cultures in (D). Error bars show the standard deviation of at least three biological replicates; the experiment was reproduced at least once. (F) Specific activities from (D) normalized to mRNA abundance from (C); error bars show the standard deviation of at least three biological replicates; the experiment was reproduced at least once; crosses indicate individual data points; comparison was performed either with minifruA (mfruA) or adjacent data (black bar) using an unpaired two-tailed t-test. ns, not significant; *, *p* < 0.05 **; *p* < 0.01 ***; *p* < 0.001.

While the results argue against the role of the sequences flanking SECIS in the 5'-UTR, they could still exert an effect on mRNA stability through base pairing. To assess this potential effect, the abundance of *bla* mRNA was determined using RT-qPCR (Figure 6E). The presence of the SECIS element in the 5'-UTR did not increase the mRNA abundance, thereby making a stabilizing effect unlikely. Strikingly, the abundance of *bla* containing the 5'-SECIS flanked with sequences complementary to *bla* (FlankBla) did not increase, as we initially hypothesized, but decreased by approximately threefold (Figure 6E). While the basis for this phenomenon is unclear, the complementary flanking sequence for the UGA-containing open reading frame may increase recoding (i.e. Sec insertion) efficiency. When the specific Bla activity was normalized to the mRNA abundance, a two-fold higher specific activity was calculated for the construct with flanking regions than for those without flanking regions (Figure 6F).

## Discussion

In this study, we investigated the *in vivo* function of methanogenic SECIS elements via targeted mutagenesis using a previously established selenium-responsive translation reporter system [20]. The data herein substantiate the notion that the KTL motif of the methanogenic SECIS is crucial element for its functionality. Other features, conserved to various degrees, such as the length of the basal stem, as well as the basal and apical GC clamps, affect SECIS function somewhat, apparently to the degree that the KTL is (de)stabilized. For example, the KTL motif in the GC2CG variant was stabilized (compared to its parent minifruA) and exhibited a higher recoding capacity, whereas a destabilized motif like in CG4AU resulted in a lower recoding capacity. Within the KTL motif, we established that, unlike for all other probed positions, the two As in the GAA of the KTL motif are absolutely invariant. KTL motif variants AAA/A and CAA/A (GAA_AAA and GAA_CAA) were found to be functional but had reduced recoding capacity. Both variants occur naturally, in the SECIS element of *fwuB* and the SECIS element for *fdhA1* in *Methanocaldococcus vulcanius* (Fig. S1B), respectively. Selenophosphate synthetase, itself a selenoprotein, is essential for selenoprotein production by providing selenophosphate [28]. It is probably no coincidence that the corresponding SECIS element (sps, Figure 2) is the most effective of those tested herein, as it would probably lead to preferential Sec insertion into SPS, thereby preserving the capacity to synthesize selenoproteins when selenium supply is limited.

**Figure 7.**
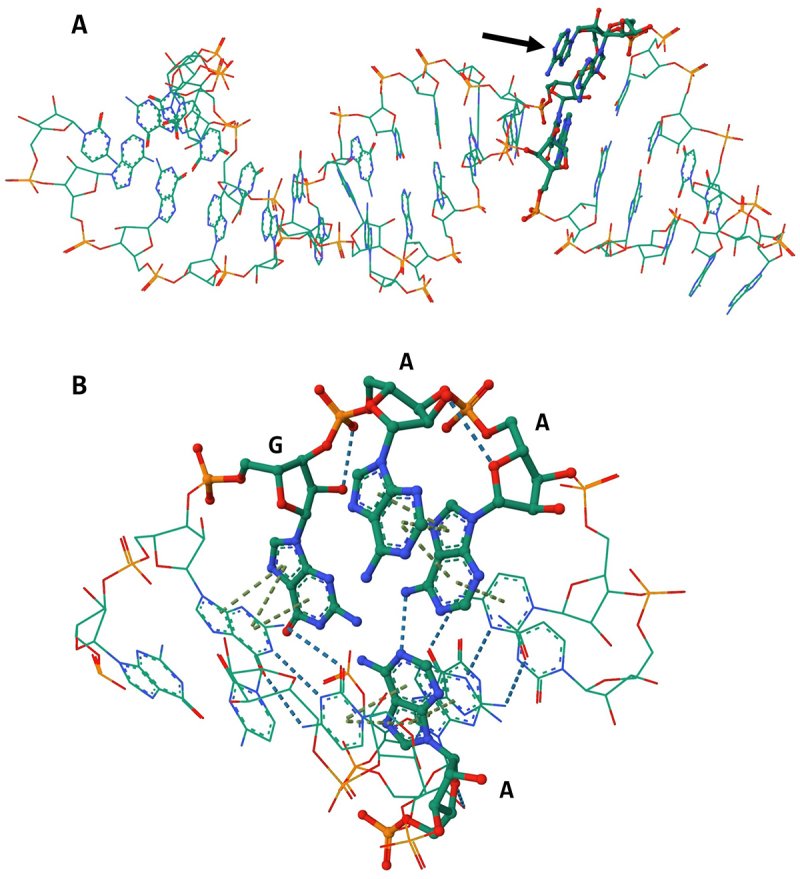
Structure model of the methanogenic SECIS element.

To better understand the internal non-covalent interactions of the *M. maripaludis* SECIS element, structural prediction was made using Alphafold3 [29,30]. The SECIS element was predicted to consist mainly of a duplex helical structure (Figure 7A). An exception is the KTL motif, which does not contain many non-covalent interactions. The central A in the GAA sequence is predicted to be displaced from the helical structure of the SECIS element, possibly due to the lack of coordination by non-covalent bonds (Figure 7B). This displacement would render the A ideally suited for interactions with binding factors.

Structure model of *fruA* SECIS element predicted with Alphafold3 [29] and visualized with Mol* [30]. The model was evaluated as confident (90 > plDDT > 70); the KTL motif is in bold font. (A) Overall structural model of *fruA* SECIS element; arrow indicates folded out A of the KTL motif. (B) Close-up view of the KTL motif and the adjacent GC clamp; dashed line indicates non-covalent interactions (hydrogen bonding or pi stacking) between the KTL motif and flanking GC pair.

The SECIS-binding factor is currently unknown in archaea. However, because communication between the site of UGA recoding (the codon in the ribosomal A site) and the signal for recoding (the SECIS in the UTR) must occur, such a factor has to exist. In bacteria, SelB is the SECIS-binding factor that interacts with bulged-out bases and with the terminal loop of the SECIS element [31,32]. In eukaryotes, the SECIS element has two conserved regions that interact with the Sec-specific elongation factor (eEFsec) and with the SECIS binding protein 2 [17]. For *M. maripaludis*, the complete exchange of the end-loop bases did not result in a complete loss of the SECIS function. In fact, only when the structure of the KTL was destroyed (excluding both As in the GAA sequence) did a variant completely lose its functionality as a SECIS. Thus, the methanogenic SECIS constituents appear to ensure the proper structure of the KTL motif. In fact, methanogenic SECIS can be shortened as long as the KTL motif is not compromised. It may be noteworthy that certain *vhuD* family SECIS elements found in Lokiarchaeota contain a motif, presumably for binding SBP2, arguing that it may represent an evolutionary transition state between SECIS elements of Archaea towards Eukarya [18]. However, to date, no SBP2 homologue has been identified in Archaea and a lokiarchaeal SECIS element is not functional in *M. maripaludis* (SLoki, Supplementary Table S2, Supplementary Fig. S1A).

When introducing SECIS variants into the 5'-UTR of the *bla* reporter, a distance of more than 60 nucleotides from the Shine-Dalgarno sequence was maintained to avoid possible effects of the KTL motif on translation initiation [33]. However, stem-loop structures, particularly those at the 5' end, have been demonstrated to significantly stabilize mRNA in *E. coli* [34]. This effect was ruled out in the present study by quantifying *bla* mRNA. The reason for the lower *bla* mRNA abundance in the presence of flanking sequences is unclear, as in other systems, the introduction of a higher degree of duplex formation (such as the flanking sequences here) stabilizes mRNA [35]. Nevertheless, it is also conceivable that the duplexes could be the target of double-strand specific RNAses, leading to reduced abundance [36,37]. Notably, while *Methanococcus voltae* appears to lack a SECIS element in the 5'-UTR of *fdhA*, it could be found in *Methanopyrus kandleri* AV19 (Table S3) without flanking complementary sequences. One could only speculate whether for a SECIS element in the 5'-UTR to function, not only its secondary structure is relevant but also other features, such as the tertiary structure of the selenoprotein mRNA or unknown factors acting in *trans*.

In conclusion, we present comprehensive experimental information for defining the methanoarchaeal SECIS element by characterizing crucial base positions (such as in the GAA sequence) and structurally important regions to preserve the KTL motif. Furthermore, demonstrating the functionality of SECIS elements in the 5'-UTR of mRNA significantly expands our knowledge of Sec recoding mechanisms in the three domains of life and opens possible avenues for the design of artificial selenoprotein genes.

## Materials and methods

### Strains and growth conditions

*Escherichia coli* DH10B was cultivated under standard conditions and transformed with plasmid DNA by electroporation as previously described [38]. Where appropriate, 100 µg ml^1^ ampicillin was added to the medium to select plasmids that confer the corresponding resistance. *M. maripaludis* strains JJ (DSMZ 2067) [39] and JH7 [23] were cultivated in a McSe medium containing 10 mM sodium acetate and 1 µM sodium selenite [21]. When selecting derivatives of self-replicating *E. coli*/*M. maripaludis* shuttle vector pWLG40NZ-R [40], 0.5 mg ml^−1^ (agar plates) or 1 mg ml^−1^ (liquid culture) neomycin were present in the medium. The cultures were pressurized with 2 × 10^5^ Pa H_2_:CO_2_ (80:20), which served as the sole energy source, and incubated at 37°C with gentle agitation. The growth of the cultures was monitored photometrically at 578 nm (OD_578_) using a Genesys 20 spectrophotometer (Thermo Fisher Scientific, USA). Transformation and plating of *M. maripaludis* were performed as previously described [28,41].

### Molecular methods and cloning

Standard molecular methods were used for plasmid manipulation [38], as listed in Table 1. All DNA fragments obtained by PCR (oligonucleotides used are listed in Table S4) and used for cloning were sequenced by Microsynth SeqLab (Göttingen, Germany) using the BigDye Terminator Cycle Sequencing protocol. The principal reporter construct (Figure 1A) has been described in detail elsewhere [20]. Briefly, it consists of (from 5' to 3', with restriction sites): XhoI, 5' region of *sla* (encoding the S-layer protein of *Methanococcus voltae* [42]), *bla* (protein variant C99U) codon-optimized for *M. maripaludis* [23], NcoI, 3'-UTR region of *M. maripaludis* JJ *fruA* (MMJJ_4570), PciI, the transcription terminator of *mcrA* of *M. voltae* [43], BglII. In pUCblaPos3 (Table 1), a nucleotide base was replaced to eliminate the interfering PciI restriction site, creating pUCblaPos3DPciI (Table 1). A specific oligonucleotide containing this base exchange sequence (Table S4) was used for PCR and subsequent Gibson Assembly [44], with pUCblaPos3 treated with the appropriate restriction enzymes (NcoI/PciI). Either pACYCblaPos3 or pUCblaPos3DPciI was used to exchange regions for the 3'-UTRs in the reporter cassette. Double-stranded oligonucleotides were used to facilitate the exchange of 3'-UTRs through restriction cloning (NcoI/PciI). To this end, two complementary oligonucleotides (Table S4) containing appropriate overhangs suitable for restriction cloning were annealed and subsequently 5' phosphorylated using T4-Polynukleotid-Kinase (Thermo Fisher Scientific, USA) after heat inactivation of the enzyme directly used for ligation. To integrate the fragments into the 5'-UTR sequence of *bla*, inverse PCR was conducted using pUCblaPos3-S, which does not carry a SECIS element, as the template and oligonucleotides with overhangs containing the SECIS element and their flanking sites (Table S4). Subsequent 5'-phosphorylation was performed using T4-Polynukleotid-Kinase (Thermo Fisher Scientific), followed by self-ligation. In this instance, only the reporter cassette was sequenced. The reporter constructs were then transferred to pWLG40NZ-R via restriction cloning using XhoI and BglII. The resulting episomal reporter plasmids (Table 1) were used to transform *M. maripaludis* JJ.

**Table 1.** Plasmids used in this study.

Name	Relevant genotype/description/construction	Reference
pACYCblaPos3	Amp^R^, P*sla-bla-*3'-UTR_*fruA*_-T_*mcrB*_ fusion from pUCblaPos3 subcloned via restriction/ligation to eliminate interfering restriction sites in the vector backbone	[20]
pACYCblaSfdh	Amp^R^, P*sla-bla-fdhA1* SECIS-T_*mcrB*_ fusion, exchange of 3'-UTR from pACYCblaPos3 with *fdhA1* SECIS	This study
pEblaminifruA	Amp^R^, Neo^R^, P*sla-bla-*3'-UTR_*fruA*_mini-T_*mcrB*_ fusion from pACYCblaminifruA in pWLG40NZ-R	[20]
pEblaSA_U	Amp^R^, P*sla-bla*-SECIS_*fruA*_GAA/U-T_*mcrB*_ fusion from pUCblaSA_U in pWLG40NZ-R	This study
pEblaSAUGC12un	Amp^R^, P*sla-bla-*SECIS_*fruA*_AUGC12unpair-T_*mcrB*_ fusion from pUCblaSAUGC12unpair in pWLG40NZ-R	This study
pEblaSCG012un	Amp^R^, P*sla-bla-*SECIS_*fruA*_CG012unpair-T_*mcrB*_ fusion from pUCblaSCG012unpair in pWLG40NZ-R	This study
pEblaSCG34AU	Amp^R^, P*sla-bla-*SECIS_*fruA*_CG34AU-T_*mcrB*_ fusion from pUCblaSCG34AU in pWLG40NZ-R	This study
pEblaSCG4AU	Amp^R^, P*sla-bla-*SECIS_*fruA*_CG4AU-T_*mcrB*_ fusion from pUCblaSCG4AU in pWLG40NZ-R	This study
pEblaSfdh	Amp^R^, Neo^R^, P*sla-bla-*3'-UTR_*fruA*_mini-T_*mcrB*_ fusion from pACYCblaSfdh in pWLG40NZ-R	This study
pEblaSfwuB	Amp^R^, P*sla-bla-fwuB* SECIS-T_*mcrB*_ fusion from pUCblaSfwuB in pWLG40NZ-R	This study
pEblaSGAA_AAA	Amp^R^, P*sla-bla-*SECIS_*fruA*_AAA/A-T_*mcrB*_ fusion from pUCblaSGAA_AAA in pWLG40NZ-R	This study
pEblaSGAA_CAA	Amp^R^, P*sla-bla-*SECIS_*fruA*_CAA/A-T_*mcrB*_ fusion from pUCblaSGAA_CAA in pWLG40NZ-R	This study
pEblaSGAA_GAG	Amp^R^, P*sla-bla-*SECIS_*fruA*_GAG/A-T_*mcrB*_ fusion from pUCblaSGAA_GAG in pWLG40NZ-R	This study
pEblaSGAA_GCA	Amp^R^, P*sla-bla-*SECIS_*fruA*_GCA/A-T_*mcrB*_ fusion from pUCblaSGAA_GCA in pWLG40NZ-R	This study
pEblaSGAA_GGA	Amp^R^, P*sla-bla-*SECIS_*fruA*_GGA/A-T_*mcrB*_ fusion from pUCblaSGAA_GGA in pWLG40NZ-R	This study
pEblaSGAA_GUA	Amp^R^, P*sla-bla-*SECIS_*fruA*_GUA/A-T_*mcrB*_ fusion from pUCblaSGAA_GUA in pWLG40NZ-R	This study
pEblaSGC12UA	Amp^R^, P*sla-bla-*SECIS_*fruA*_GC12UA-T_*mcrB*_ fusion from pUCblaSGC12UA in pWLG40NZ-R	This study
pEblaSGC12un	Amp^R^, P*sla-bla-*SECIS_*fruA*_GC12unpair-T_*mcrB*_ fusion from pUCblaSGC12unpair in pWLG40NZ-R	This study
pEblaSGC2CG	Amp^R^, P*sla-bla*-SECIS_*fruA*_GC2CG-T_*mcrB*_ fusion from pUCblaSGC2CG in pWLG40NZ-R	This study
pEblaSLoki	Amp^R^, P*sla-bla-*Loki*vhuU* SECIS-T_*mcrB*_ fusion from pUCblaSLoki in pWLG40NZ-R	This study
pEblaSsps	Amp^R^, P*sla-bla-sps* SECIS-T_*mcrB*_ fusion from pUCblaSsps in pWLG40NZ-R	This study
pEblaSTLoopGGCGA	Amp^R^, P*sla-bla-* SECIS_*fruA*_TLoopGGCGA -T_*mcrB*_ fusion from pUCblaSTLoopGGCGA in pWLG40NZ-R	This study
pEbla5GC2CGfBla	Amp^R^, P*sla-*SECIS_*fruA*_GC2CGfBla-*bla-*T_*mcrB*_ fusion from pUCbla5GC2CGfBla in pWLG40NZ-R	This study
pEbla5GC2CGfFdh	Amp^R^, P*sla-*SECIS_*fruA*_GC2CGfFdh-*bla*-T_*mcrB*_ fusion from pUCbla5GC2CGfFdh in pWLG40NZ-R	This study
pUCblaPos3	pUC57-BsaI-free, P*sla-bla-*3'-UTR_*fruA*_-T_*mcrB*_ fusion with codon exchange at Position 3 (297C→A), general cloning	[20]
pUCblaPos3DPciI	Amp^R^, P*sla-bla-*3'-UTR_*fruA*_-T_*mcrB*_, pUCblaPos3, interfering PciI eliminated in the vector backbone	This study
pUCblaPos3-S	Amp^R^, P*sla-bla-*T_*mcrB*_ fusion, UTR-encoding sequence from pUCblaPos3 excised (NcoI, PciI) and re-ligated, general cloning	[20]
pUCblaSA_U	Amp^R^, P*sla-bla*-SECIS_*fruA*_GAA/U-T_*mcrB*_ fusion, exchange of 3'-UTR from pUCblaPos3DPciI with SECIS_*fruA*_GAA/U	This study
pUCblaSAUGC12unpair	Amp^R^, P*sla-bla-*SECIS_*fruA*_AUGC12unpair-T_*mcrB*_ fusion, exchange of 3'-UTR from pUCblaPos3DPciI with SECIS_*fruA*_AUGC12unpair	This study
pUCblaSCG012unpair	Amp^R^, P*sla-bla-*SECIS_*fruA*_CG012unpair-T_*mcrB*_ fusion, exchange of 3'-UTR from pUCblaPos3DPciI with SECIS_*fruA*_CG012unpair	This study
pUCblaSCG34AU	Amp^R^, P*sla-bla-*SECIS_*fruA*_CG34AU-T_*mcrB*_ fusion, exchange of 3'-UTR from pUCblaPos3DPciI with SECIS_*fruA*_CG34AT	This study
pUCblaSCG4AU	Amp^R^, P*sla-bla-*SECIS_*fruA*_CG4AU-T_*mcrB*_ fusion, exchange of 3'-UTR from pUCblaPos3DPciI with SECIS_*fruA*_CG4AT	This study
pUCblaSfwuB	Amp^R^, P*sla-bla-fwuB* SECIS-T_*mcrB*_ fusion, exchange of 3'-UTR from pUCblaPos3DPciI with *fwuB* SECIS	This study
pUCblaSGAA_AAA	Amp^R^, P*sla-bla-*SECIS_*fruA*_AAA/A-T_*mcrB*_ fusion, exchange of 3'-UTR from pUCblaPos3DPciI with SECIS_*fruA*_AAA/A	This study
pUCblaSGAA_CAA	Amp^R^, P*sla-bla-*SECIS_*fruA*_CAA/A-T_*mcrB*_ fusion, exchange of 3'-UTR from pUCblaPos3DPciI with SECIS_*fruA*_CAA/A	This study
pUCblaSGAA_GAG	Amp^R^, P*sla-bla-*SECIS_*fruA*_GAG/A-T_*mcrB*_ fusion, exchange of 3'-UTR from pUCblaPos3DPciI with SECIS_*fruA*_GAG/A	This study
pUCblaSGAA_GCA	Amp^R^, P*sla-bla-*SECIS_*fruA*_GCA/A-T_*mcrB*_ fusion, exchange of 3'-UTR from pUCblaPos3DPciI with SECIS_*fruA*_GCA/A	This study
pUCblaSGAA_GGA	Amp^R^, P*sla-bla-*SECIS_*fruA*_GGA/A-T_*mcrB*_ fusion, exchange of 3'-UTR from pUCblaPos3DPciI with SECIS_*fruA*_GGA/A	This study
pUCblaSGAA_GUA	Amp^R^, P*sla-bla-*SECIS_*fruA*_GUA/A-T_*mcrB*_ fusion, exchange of 3'-UTR from pUCblaPos3DPciI with SECIS_*fruA*_GUA/A	This study
pUCblaSGC12UA	Amp^R^, P*sla-bla-*SECIS_*fruA*_GC12UA-T_*mcrB*_ fusion, exchange of 3'-UTR from pUCblaPos3DPciI with SECIS_*fruA*_GC12TA	This study
pUCblaSGC12unpair	Amp^R^, P*sla-bla-*SECIS_*fruA*_GC12unpair-T_*mcrB*_ fusion, exchange of 3'-UTR from pUCblaPos3DPciI with SECIS_*fruA*_GC12unpair	This study
pUCblaSGC2CG	Amp^R^, P*sla-bla*-SECIS_*fruA*_GC2CG-T_*mcrB*_ fusion, exchange of 3'-UTR from pUCblaPos3DPciI with SECIS_*fruA*_GC2CG	This study
pUCblaSLoki	Amp^R^, P*sla-bla-*Loki*vhuU* SECIS-T_*mcrB*_ fusion, exchange of 3'-UTR from pUCblaPos3DPciI with metagenome Lokiarchaea *vhuU*.2 secIS	This study
pUCblaSsps	Amp^R^, P*sla-bla-sps* SECIS-T_*mcrB*_ fusion, exchange of 3'-UTR from pUCblaPos3DPciI with *sps* SECIS	This study
pUCblaSTLoopGGCGA	Amp^R^, P*sla-bla-* SECIS_*fruA*_TLoopGGCGA -T_*mcrB*_ fusion, exchange of 3'-UTR from pUCblaPos3DPciI with SECIS_*fruA*_TerminalLoopGGCGA	This study
pUCbla5GC2CGfBla	Amp^R^, P*sla-*SECIS_*fruA*_GC2CGfBla-*bla-*T_*mcrB*_ fusion, insert in 5'-UTR from pUCblaPos3-S the SECIS_*fruA*_GC2CG with flanking sites complementary to the ORF of Bla 3' of UGA	This study
pUCbla5GC2CGfFdh	Amp^R^, P*sla-*SECIS_*fruA*_GC2CGfFdh-*bla*-T_*mcrB*_ fusion, insert in 5'-UTR from pUCblaPos3-S the SECIS_*fruA*_GC2CG with flanking sites not complementary to the ORF of Bla 3' of UGA	This study
pUC57-BsaI-free	Amp^R^, general cloning	Biocat.com
pWLG40NZ-R	Amp^R^, Neo^R^, *E. coli*/*M. maripaludis* shuttle vector	[40]

### Quantification of beta-lactamase

The beta-lactamase (Bla) activity in *M. maripaludis* JJ carrying the reporter plasmids was quantified using nitrocefin (Biomol, Hamburg, Germany), as described previously [23], with the exception that the cells were harvested at an OD_578_ of approximately 0.3. Bla activity (data summarized in Table S2) was determined at 486 nm using a molar extinction coefficient of 20,500 M^−1^ cm^−1^ and expressed as milliunits (mU) per milligram protein (where 1 U = 1 μmol nitrocefin cleaved per min). The values in question must be multiplied by 0.016 to convert mU into SI unit nkat. Proteins in cell fractions were determined using the Bradford method [45], with bovine serum albumin used as the standard.

### Quantification of mRNA

*M. maripaludis* mRNA was quantified via reverse transcription (RT) coupled to quantitative PCR (qPCR), as previously described [20]. RNA was isolated using the High Pure RNA Tissue Kit (Roche, Mannheim, Germany) according to the manufacturer’s instructions. RNA preparations were employed for the synthesis of cDNA with gene-specific oligonucleotides (Table S4), and the absence of DNA was confirmed via qPCR. The synthesis of cDNA, qPCR, and data analysis were conducted as described previously [22], except that for cDNA synthesis, SuperScript III Reverse Transcriptase (Invitrogen, Carlsbad, USA) was used, and for qPCR, the Luna Universal qPCR Master Mix (New England Biolabs, Ipswitch, USA) was used in conjunction with qTOWER^3^ (Analytik Jena, Jena, Germany). For cDNA synthesis of *mcrB*, which encodes the β-subunit of methyl-coenzyme M reductase, the oligonucleotide used was specific for the allele from strain S2 (MMP1555, Table S4) [22] and differs from the corresponding sequence of strain JJ (MMJJ_2810) by a single base. The data were analysed and normalized to the expression of *mcrB* as previously described [22]. The amount of mRNA *bla* copy numbers are shown per mRNA copy number of *mcrB* (data summarized in Table S5).

### Structure prediction

The minimal free energy (MFE) predictions of RNA structures were conducted using the RNAfold algorithm [25]. The MFE and percentage of frequencies for the predictions of SECIS variants are presented in Table S1. The MFE predictions of complete mRNAs were performed for *fdhA* gene loci from Methanococccales (Table S3), assuming a transcriptional start of 300 nucleotides 5' of the translational start. The number of nucleotides in the flanking regions of the SECIS elements was manually extracted from the MFE predictions of complete *fdhA* mRNAs.

Three-dimensional structural models were predicted using Alphafold3 [29] and visualized using Mol* [30]. The models were evaluated as confident (90 > plDDT > 70).

### Statistical analysis

A two-tailed unpaired t-test was used to compare the two cohorts, and additional statistical analyses were conducted using GraphPad Prism version 5.03 (GraphPad Software, San Diego, USA).

## Supplementary Material

NP244393123_Suppl_rev_marked pre export.docx

## Data Availability

All data and material reported here are available upon reasonable request, which should be directed to the corresponding author.

## Writing Assistance

The text was written with the assistance of DeepL Write.
