# Supplementary material for: Defining the methanogenic SECIS element in vivo by targeted mutagenesis
Source: RNA Biol. 2025 Feb 25;22(1):1–13. doi: 10.1080/15476286.2025.2472448 (PMC11881835; doi:10.1080/15476286.2025.2472448)
Supplement: NP244393123_Suppl_rev_marked pre export.docx [file KRNB_A_2472448_SM4030.docx]

**Supplementary Material**

for

Defining the methanogenic SECIS Element *in vivo* by targeted mutagenesis

Nils Peiter (ORCID ID 0000-0002-0181-6605), Anna Einert, Pauline Just, Frida Jannasch (ORCID ID 0009-0000-7185-436X), Marija Najdovska and Michael Rother (ORCID ID 0000-0002-2306-8783)*

Fakultät Biologie, Technische Universität Dresden, 01062 Dresden, Germany

This file includes:

Supplementary Figures S1

Supplementary Tables S1-S6


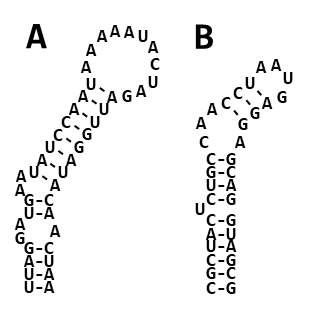


Supplementary Figure S1: RNA structure prediction from (A) the SECIS element vhuU.2 of a Lokiarchaea adapted from [18] and (B) of the SECIS element of *fdhA1* from *Methanocaldococcus vulcanius* M7. Minimum free energy RNA-structure were predicted using RNAfold [44]; dashes indicate Watson-Crick base pairing

Supplementary Table S1: Minimal free energy and frequency of structure predictions with RNAfold in this study

| Construct^a^ | Minimal free energy^b^ | Frequency^c^ |
| --- | --- | --- |
| A_U | -12.14 | 35.27 |
| AUGC12un | -3.05 | 52.13 |
| CG012un | -5.10 | 86.72 |
| CG34AU | -8.70 | 27.27 |
| CG4AU | -7.40 | 58.42 |
| fdhA1 | -11.55 | 66.36 |
| fwuB | -8.09 | 86.44 |
| GAA_AAA | -9.58 | 88.46 |
| GAA_CAA | -9.58 | 88.24 |
| GAA_GAG | -9.72 | 70.32 |
| GAA_GCA | -9.75 | 66.75 |
| GAA_GGA | -9.60 | 84.90 |
| GAA_GUA | -9.60 | 84.47 |
| GC12UA | -5.56 | 76.94 |
| GC12un | -2.71 | 43.56 |
| GC2CG | -9.70 | 84.91 |
| Loki | -6.90 | 20.91 |
| minifruA | −9.5 | 71.2 |
| sps | -9.25 | 56.77 |
| TLoop | -9.90 | 72.64 |

a: see Table S1

b: kcal mol^−1^

c: in %

Supplementary Table S2: Reporter activity measurements in *M. maripaludis* JJ or JH7

| Plasmid^a^ | Construct | #Replicates | Bla activity^b^ | ± Standard deviation |
| --- | --- | --- | --- | --- |
| pEblaminifruA^d^ | minifruA | 4 | 426^d^ | 47^d^ |
| pEblaminifruA^c^ |  | 4 | 368 | 39 |
| pEblaPos3-S^d^ | -S | 4 | 22.1 | 5.1 |
| pEblaSA_U | A_U | 4 | 46 | 15 |
| pEblaSAUGC12un | AUGC12un | 5 | 75.8 | 4.4 |
| pEblaSCG012un | CG012un | 5 | 117 | 24 |
| pEblaSCG34AU | CG34AU | 4 | 37 | 18 |
| pEblaSCG4AU | CG4AU | 5 | 179 | 16 |
| pEblaSfdh | fdhA1 | 4 | 428 | 39 |
| pEblaSfwuB | fwuB | 5 | 232 | 29 |
| pEblaSGAA_AAA | GAA_AAA | 5 | 298 | 42 |
| pEblaSGAA_CAA | GAA_CAA | 5 | 97 | 18 |
| pEblaSGAA_GAG | GAA_GAG | 4 | 22.6 | 1.9 |
| pEblaSGAA_GCA | GAA_GCA | 4 | 30.9 | 4.0 |
| pEblaSGAA_GGA | GAA_GGA | 5 | 13.5 | 2.1 |
| pEblaSGAA_GUA | GAA_GUA | 5 | 17.5 | 4.0 |
| pEblaSGC12UA | GC12UA | 5 | 257 | 27 |
| pEblaSGC12un | GC12un | 4 | 21.0 | 1.0 |
| pEblaSGC2CG | GC2CG | 13 | 655 | 147 |
| pEblaSLoki | SLoki | 5 | 21.1 | 6.4 |
| pEblaSsps | sps | 5 | 699 | 46 |
| pEblaSTLoopGGCGA | TLoop | 5 | 172 | 18 |
| pEbla5GC2CGfBla | FlankBla | 9 | 405 | 75 |
| pEbla5GC2CGfFDH | FlankFdh | 9 | 385 | 77 |

a: see Table 1

b: mU mg^-1^, mean

c: in strain JH7

d: in strain JJ [20]

Supplementary Table S3: Amount of duplex formation for *fdhA1* in different species of the Methanococcales.

| Organism | Locus Tag | Length of the flanking sites upstream and downstream of the SECIS^a^ | NCBI Reference Sequence |
| --- | --- | --- | --- |
| *Methanocalcoccocus jannaschii* JAL-1 | MJ_RS07235 | 9 and 16 | NC_000909 |
| *Methanocaldococcus* *bathoardescens* JH146 | JH146_RS01240 | 11 und 17 | NZ_CP009149 |
| *Methanocaldococcus fervens* AG86 | MEFER_RS03285 | 7 and 17 | NC_013156 |
| *Methanocaldococcus vulcanius* M7 | METVU_RS05695 | 6 and 15 | NC_013407 |
| *Methanococcus aeolicus* Nankai-3 | MAEO_RS02870 | 7 and 13 | NC_009635 |
| *Methanococcus aeolicus* PL15/HP | N6C89_RS05245 | 7 and 13 | NZ_CP104873 |
| *Methanococcus maripaludis* C5 | MMARC5_RS01500 | 7 and 8 | NC_009135 |
| *Methanococcus maripaludis* JJ | MMJJ_RS07665 | 7 and 10 | NZ_CP026606 |
| *Methanococcus maripaludis* KA1 | MMKA1_RS07645 | 7 and 10 | NZ_AP011526 |
| *Methanococcus maripaludis* OS7 | MMOS7_RS07135 | 7 and 10 | NZ_AP011528 |
| *Methanococcus maripaludis* S2 | locus_tag: MMP_RS06685 | No | NC_005791 |
| *Methanococcus maripaludis* X1 | locus_tag: GYY_RS07170 | No | NC_015847 |
| *Methanococcus vannielii* SB | MEVAN_RS03105 | 9 and 11 | NC_009634 |
| *Methanococcus voltae* PS Ga0416752_04 | M2325_001473  M2325_000557 | No | NZ_JANUCQ010000004  NZ_JANUCQ010000001 |
| *Methanococcus voltae* C2 | J2127_001280  J2127_001398 | No | NZ_JAGGMO010000011  NZ_JAGGMO010000010 |
| *Methanopyrus kandleri* AV19 | MK_RS09240 | No | NC_003551 |
| *Methanopyrus* sp. KOL6 | BW919_RS06955 | No | NZ_CP019470 |
| *Methanopyrus* sp. SNP6 | BW921_RS07675 | No | NZ_CP019436 |
| *Methanothermococcus okinawensis* IH1 | METOK_RS06245 | 6 and 17 | NC_015636 |
| *Methanothermococcus thermolithotrophicus* SN-1 | OGY79_RS01065 | 8 and 17 | NZ_OX296583 |
| *Methanotorris formicicus* Mc-S-70 | MetfoDRAFT_1255 | 14 and 13 | AGJL01000030 |
| *Methanotorris igneus* Kol 5 | METIG_RS03755 | 9 and 16 | NC_015562 |

a: Number of duplex formed

Supplementary Table S4: Oligonucleotides used in this study.

| Name | Sequence 5’🡪3’ (restriction sites/ overhangs are underlined) | Use |
| --- | --- | --- |
| o_bla_qPCR_fw3 | CAGACAACACAGCAGCAAAC | qPCR of *bla* and sequencing of 3’-UTR elements |
| o_bla_qPCR_rev2 | TGCTGCGATGATTCCTCTTG | cDNA synthesis of *bla* |
| o_bla_qPCR_rev3 | TGTGTCTCTTTCGTCGTTAGGG | qPCR of *bla* and sequencing of 5’-UTR elements |
| o1555cDNA-2^a^ | GATTACGCCGTCAGCAAT**A**GC | cDNA synthesis of *mcrB*,  base different in JJ (G) compared to S2 (A) in bold |
| o1555RTFor^a^ | CAGTTAACCACGCAGTTGC | qPCR of *mcrB* |
| o1555RTRev^a^ | GTTATCAGCGTTTAATCCTTGG | qPCR of *mcrB* |
| oA_Ufor_JJfruASECIS | CATGGGTCTTGGAAAGGgaaCCTTAATGG**t**CCTTGAAAGACA | Cloning of SECIS*_fruA_*GAA/U element, base exchange in bold |
| oA_Urev_JJfruASECIS | CATGTGTCTTTCAAGG**a**CCATTAAGGttcCCTTTCCAAGACC | Cloning of SECIS*_fruA_*GAA/U element, base exchange in bold |
| oAUGC_12unfor | CATGG**aacacacac**AGGgaaCCTTAATGGaCCT**cacacaca**A | Cloning of SECIS*_fruA_*AUGC12unpair element, base exchange in bold |
| oAUGC_12unrev | CATGT**tgtgtgtg**AGGtCCATTAAGGttcCCT**gtgtgtgtt**C | Cloning of SECIS*_fruA_*AUGC12unpair element, base exchange in bold |
| oCG_012unfor | CATGG**aacacacaaCCC**gaaCCTTAATGGa**GGGaacacaca**A | Cloning of SECIS*_fruA_*CG012unpair element, base exchange in bold |
| oCG_012unrev | CATGT**tgtgtgttCCC**tCCATTAAGGttc**GGGttgtgtgtt**C | Cloning of SECIS*_fruA_*CG012unpair element, base exchange in bold |
| oCG_34_AUforJJSECIS | CATGGGTCTTGGAAAGGgaa**aa**TTAAT**tt**aCCTTGAAAGACA | Cloning of SECIS*_fruA_*CG34AU element, base exchange in bold |
| oCG_34_AUrevJJSECIS | CATGTGTCTTTCAAGGt**aa**ATTAAtt**tt**cCCTTTCCAAGACC | Cloning of SECIS*_fruA_*CG34AU element, base exchange in bold |
| oCG_4_AUfor_JJSECIS | CATGGGTCTTGGAAAGGgaaC**a**TTAAT**t**GaCCTTGAAAGACA | Cloning of SECIS*_fruA_*CG4AU element, base exchange in bold |
| oCG_4_AUrev_JJSECIS | CATGTGTCTTTCAAGGtC**a**ATTAA**t**GttcCCTTTCCAAGACC | Cloning of SECIS*_fruA_*CG4AU element, base exchange in bold |
| oGA_blaWLNZR_For | TCTCTTCTTCTTCAGGGAGC**TCGAGTTTGTAAAGTGGTAG** | Gibson cloning of reporter into pWLG40NZ-R, overlap for reporter construct in bold |
| oGA_blaWLNZR_rev | GGCGTTTTTTATGACCTACA**GATCTAATCAATTTTTAAAAATATATAAAAAAAGG** | Gibson cloning of reporter into pWLG40NZ-R, overlap with reporter construct in bold |
| oGAA_AAAfor_JJfruASECIS | CATGGGTCTTGGAAAGG**a**aaCCTTAATGGaCCTTGAAAGACA | Cloning of SECIS*_fruA_*AAA/A element, base exchange in bold |
| oGAA_AAArev_JJfruASECIS | CATGTGTCTTTCAAGGtCCATTAAGGtt**t**CCTTTCCAAGACC | Cloning of SECIS*_fruA_*AAA/A element, base exchange in bold |
| oGAA_CAAfor_JJfruASECIS | CATGGGTCTTGGAAAGG**c**aaCCTTAATGGaCCTTGAAAGACA | Cloning of SECIS*_fruA_*CAA/A element, base exchange in bold |
| oGAA_CAArev_JJfruASECIS | CATGTGTCTTTCAAGGtCCATTAAGGtt**g**CCTTTCCAAGACC | Cloning of SECIS*_fruA_*CAA/A element, base exchange in bold |
| oGAA_GAGfor_JJSECIS | CATGGGTCTTGGAAAGGga**g**CCTTAATGGaCCTTGAAAGACA | Cloning of SECIS*_fruA_*GAG/A element, base exchange in bold |
| oGAA_GAGrev_JJSECIS | CATGTGTCTTTCAAGGtCCATTAAGG**c**tcCCTTTCCAAGACC | Cloning of SECIS*_fruA_*GAG/A element, base exchange in bold |
| oGAA_GCAfor_JJSECIS | CATGGGTCTTGGAAAGGg**c**aCCTTAATGGaCCTTGAAAGACA | Cloning of SECIS*_fruA_*GCA/A element, base exchange in bold |
| oGAA_GCAfrev_JJSECIS | CATGTGTCTTTCAAGGtCCATTAAGGt**g**cCCTTTCCAAGACC | Cloning of SECIS*_fruA_*GCA/A element, base exchange in bold |
| oGApuc57delPciIfor | ggaccttgaaagacacaaattaacttac | Gibson cloning for one base exchange to delete interfering PciI site in pUCblaPos3 |
| oGApuc57delPciIrev | ctggccttttgctca**A**atgttctttc | Gibson cloning for one base exchange to delete interfering PciI site in pUCblaPos3, base exchange in bold |
| oGC_12_UAforJJSECIS | CATGGGTCTTGGAAA**tt**gaaCCTTAATGGa**aa**TTGAAAGACA | Cloning of SECIS*_fruA_*GC12UA element, base exchange in bold |
| oGC_12_UArevJJSECIS | CATGTGTCTTTCAA**tt**tCCATTAAGGttc**aa**TTTCCAAGACC | Cloning of SECIS*_fruA_*GC12UA element, base exchange in bold |
| oGC_12unfor | CATGG**aacacacaca**GGgaaCCTTAATGGaCC**acacacaca**A | Cloning of SECIS*_fruA_*GC12unpair element, base exchange in bold |
| oGC_12unrev | CATGT**tgtgtgtgt**GGtCCATTAAGGttcCC**tgtgtgtgtt**C | Cloning of SECIS*_fruA_*GC12unpair element, base exchange in bold |
| oGC_2_CGfor_JJSECIS | CATGGGTCTTGGAAAG**c**gaaCCTTAATGGa**g**CTTGAAAGACA | Cloning of SECIS*_fruA_*GC2CG element, base exchange in bold |
| oGC_2_CGrev_JJSECIS | CATGTGTCTTTCAAG**c**tCCATTAAGGttc**g**CTTTCCAAGACC | Cloning of SECIS*_fruA_*GC2CG element, base exchange in bold |
| oGC2CG_GAA_GGAfor | CATGGGTCTTGGAAAG**c**g**g**aCCTTAATGGa**g**CTTGAAAGACA | Cloning of SECIS*_fruA_*GGA/A element, base exchange in bold |
| oGC2CG_GAA_GGArev | CATGTGTCTTTCAAG**c**tCCATTAAGGt**c**c**g**CTTTCCAAGACC | Cloning of SECIS*_fruA_*GGA/A element, base exchange in bold |
| oGC2CG_GAA_GUAfor | CATGGGTCTTGGAAAG**c**g**t**aCCTTAATGGa**g**CTTGAAAGACA | Cloning of SECIS*_fruA_*GUA/A element, base exchange in bold |
| oGC2CG_GAA_GUArev | CATGTGTCTTTCAAG**c**tCCATTAAGGt**a**c**g**CTTTCCAAGACC | Cloning of SECIS*_fruA_*GUA/A element, base exchange in bold |
| oGC2CGflankBla5UTRfor | ATGGAgCTTGAAAGACcctaggATAAGTTTGC**ATACAAAAATAAAAACAAAAGGTGCATTACAATGG** | Cloning of SECIS*_fruA_*GC2CG with flanking sites complementary to the ORF of Bla 3’ of UGA, overlap with reporter construct in bold |
| oGC2CGflankBla5UTRrev | TAAGGTTCgCTTTCCAAGACccatggTATTAACAAC**TATTTTTTAGGTTTTGTATTTCCGGTAGTAATCAATAAGTTG** | Cloning of SECIS*_fruA_*GC2CG with flanking sites complementary to the ORF of Bla 3’ of UGA, overlap with reporter construct in bold |
| oGC2CGflankFdh5UTRfor | ATGGAgCTTGAAAGACcctaggttgaaccaaa**ATACAAAAATAAAAACAAAAGGTGCATTACAATGG** | Cloning of SECIS*_fruA_*GC2CG with flanking sites not complementary to the ORF of Bla 3’ of UGA, overlap with reporter construct in bold |
| oGC2CGflankFdh5UTRrev | TAAGGTTCgCTTTCCAAGACccatggtttgtgctat**TATTTTTTAGGTTTTGTATTTCCGGTAGTAATCAATAAGTTG** | Cloning of SECIS*_fruA_*GC2CG with flanking sites not complementary to the ORF of Bla 3’ of UGA, overlap with reporter construct in bold |
| opWLG40-For | CATTGTTAGACCTGCGACAG | Sequencing of reporter in pWLG40NZ-R |
| oTLoopGGCGAfor | CATGGGTCTTGGAAAGGgaaCC**GGCGA**GGaCCTTGAAAGACA | Cloning of SECIS*_fruA_*TerminalLoopGGCGA element, base exchange in bold |
| oTLoopGGCGArev | CATGTGTCTTTCAAGGtCC**TCGCC**GGttcCCTTTCCAAGACC | Cloning of SECIS*_fruA_*TerminalLoopGGCGA element, base exchange in bold |

a: [22]

Supplementary Table S5: mRNA abundance in *M. maripaludis* JJ constructs

| Plasmid^a^ | Construct | #Replicates | *bla* copy number per *mcrB* copy number^b^ | ± Standard deviation^c^ | Bla^b^ activity per (copy number per *mcrB* copy number) | ± Standard deviation^d^ |
| --- | --- | --- | --- | --- | --- | --- |
| pEbla5GC2CGfBla | FlankBla | 4 | 4.1 | 1.3 | 121 | 25 |
| pEbla5GC2CGfFDH | FlankFdh | 5 | 14.0 | 6.4 | 35 | 19 |
| pEblaSGC2CG | GC2CG | 3 | 13.1 | 3.7 | 43.9 | 9.6 |

a: see Table 1

b: mean

c: ± Standard deviation of mean *bla* copy number per *mcrB* copy number

d: ± Standard deviation of mean Bla activity per (copy number per *mcrB* copy number)
